# Rapid enhancement of touch from non-informative vision of the hand

**DOI:** 10.1016/j.neuropsychologia.2012.04.020

**Published:** 2012-07

**Authors:** Flavia Cardini, Matthew R. Longo, Jon Driver, Patrick Haggard

**Affiliations:** a Centre for Studies and Researches in Cognitive Neuroscience (CsrNC), University of Bologna, Via Riccardo Brusi 20, 47023 Cesena, Italy; b Institute of Cognitive Neuroscience, University College London, UK; c Department of Psychological Sciences, Birkbeck, University of London, UK; d Wellcome Centre for Neuroimaging, UCL, UK

**Keywords:** Event related potentials, Multisensory interaction, Somatosensory processing, Visual enhancement of touch

## Abstract

Processing in one sensory modality may modulate processing in another. Here we investigate how simply viewing the hand can influence the sense of touch. Previous studies showed that non-informative vision of the hand enhances tactile acuity, relative to viewing an object at the same location. However, it remains unclear whether this Visual Enhancement of Touch (VET) involves a phasic enhancement of tactile processing circuits triggered by the visual event of seeing the hand, or more prolonged, tonic neuroplastic changes, such as recruitment of additional cortical areas for tactile processing. We recorded somatosensory evoked potentials (SEPs) evoked by electrical stimulation of the right middle finger, both before and shortly after viewing either the right hand, or a neutral object presented via a mirror. Crucially, and unlike prior studies, our visual exposures were unpredictable and brief, in addition to being non-informative about touch. Viewing the hand, as opposed to viewing an object, enhanced tactile spatial discrimination measured using grating orientation judgements, and also the P50 SEP component, which has been linked to early somatosensory cortical processing. This was a trial-specific, phasic effect, occurring within a few seconds of each visual onset, rather than an accumulating, tonic effect. Thus, somatosensory cortical modulation can be triggered even by a brief, non-informative glimpse of one's hand. Such rapid multisensory modulation reveals novel aspects of the specialised brain systems for functionally representing the body.

## Highlights

► Vision of the body modulates touch and somatosensory evoked potentials. ► Previous studies blocked trials, making the rapidity of this effect unclear. ► Brief, event-related vision of the hand or non-body object. ► Vision of the hand rapidly enhances tactile acuity and the P50 SEP component.

## 1 Introduction

### 1.1 Two timescales for multisensory interaction

Strong multisensory interactions exist between vision and touch. Studies in humans have emphasised perceptual (Ernst & Banks, 2002) or attentional (Driver & Grossenbacher, 1996) links that relate visual and tactile information to improve multisensory representation of a common stimulus object. Single-unit recordings in animals have emphasised spatial overlap between visual and tactile receptive fields (RFs) of bimodal neurons in association areas of cortex, notably premotor and parietal areas (Graziano, Yap, & Gross, 1994). Findings that the visual RFs can shift to follow the hand have been taken to suggest a function of these neurons in monitoring peripersonal space around the hand (Graziano & Cooke, 2006). Importantly, the visual-tactile interactions in parietal bimodal neurons are highly temporally specific: information from the two modalities must arrive within a narrow time-window of a few hundred milliseconds in order for these multisensory neurons to integrate their various inputs (Avillac, Ben Hamed, & Duhamel, 2007) thereby creating a unified percept (Di Luca, Machulla, & Ernst, 2009). This accords with the notion that temporal summation of action potentials from lower-level unisensory areas onto higher order neurons plays an important role in multisensory interactions at the millisecond timescale (Stein, Meredith, & Wallace, 1993).

A second distinct class of multisensory interactions involves more tonic neuroplastic changes in representations within sensory areas. One striking example of neuroplastic change is the unmasking of latent connections between different sensory cortices following sensory deprivation. Facchini and Aglioti found that light-depriving healthy volunteers for 90 min increased their tactile acuity, perhaps because absence of visual signals allowed visual cortex to be activated for tactile processing by latent somatosensory inputs (Facchini & Aglioti, 2003). The potential access of tactile signals to visual cortex is further supported by functional imaging studies in healthy volunteers (Sathian & Zangaladze, 2002), and by findings that visual cortex is recruited during tactile Braille reading in the blind (Sadato et al., 1996), and even in blindfolded volunteers (Merabet et al., 2008). Finally, such neuroplastic changes in cortical processing may involve Hebbian associative processes: repeated paired stimulation within a single modality (Hodzic, Veit, Karim, Erb, & Godde, 2004; Stavrinou et al., 2007), or cross-modal pairing between vision and touch (Schaefer, Flor, Heinze, & Rotte, 2006; Zhou & Fuster, 2000) can lead to changes in somatosensory cortical representation and improved tactile perception. In summary, tonic neuroplastic changes can underlie some visual-tactile interactions. Such multisensory neuroplasticity will take place over timescales from minutes up to the whole lifespan (Merabet & Pascual-Leone, 2010), quite unlike the more ‘phasic’ type of multisensory interaction we described earlier.

### 1.2 Visual enhancement of touch

We have identified one form of visual-tactile interaction behaviourally in humans: simply viewing the body improves tactile perception within the viewed skin region, relative to viewing an object in the same location (Kennett, Taylor-Clarke, & Haggard, 2001). Importantly, this *Visual Enhancement of Touch* (VET) does not involve standard feedforward convergence of visual and tactile information about a common object, since it can even occur when vision is entirely non-informative about touch, for example when the tactile stimulation itself cannot be seen (Taylor-Clarke, Kennett, & Haggard, 2004). Instead, viewing the body seems to provide a visual context that modulates tactile processing. Moreover electroencephalographic (EEG) and Transcranial Magnetic Stimulation (TMS) studies suggest that the visual context of seeing the body can influence processing in early somatosensory cortex (Cardini, Longo, & Haggard, 2011; Fiorio & Haggard, 2005; Longo, Betti, Aglioti, & Haggard, 2009; Longo, Iannetti, Mancini, Driver, & Haggard, 2012a; Longo, Pernigo, & Haggard, 2011; Taylor-Clarke, Kennett, & Haggard, 2002). In particular, viewing the body appears to preset the tactile circuits involved in tactile discrimination, perhaps via top-down links from visual or multisensory areas into somatosensory cortex.

The timing of such modulatory effects has not been studied, yet is theoretically important. It remains unclear if they resemble fast mechanisms of phasic multisensory integration operating over seconds, or slower tonic mechanisms of neuroplastic change operating over minutes or hours. One VET study showed that viewing the hand enhanced tactile acuity after a 2 s dark interval, and to a lesser, but still-significant degree after 10 s (Taylor-Clarke et al., 2004). This study implies that VET can *persist* for at least 10 seconds. However, either event-related, phasic multisensory integration of a body context, or slower neuroplastic changes could potentially have effects persisting over this timescale. Perhaps the most critical distinction between the two potential types of mechanism arises in the time taken for the VET effect to *develop* from the moment the body is viewed. Here, the two multisensory mechanisms outlined above make different predictions. If VET reflects phasic integration of visual context with incoming tactile information, it should emerge very rapidly after viewing the body. On the other hand, if VET depends on plastic reorganisation of visual-tactile links following prolonged co-occurrence of touch with vision of the body, then presumably it should require at least some minutes (Facchini & Aglioti, 2003). To date, the time required for VET to develop has not been investigated.

Investigating this issue would clearly require an event-related rather than a blocked design. In previous studies, the effects of viewing the body or a neutral object were contrasted across separate blocks, each lasting several minutes (Kennett et al., 2001; Taylor-Clarke et al., 2002; Cardini et al., 2011). In blocked designs, vision might influence touch either through fast or slow mechanisms or both. Studies of VET using event-related potentials (Cardini et al., 2011; Taylor-Clarke et al., 2002) nevertheless still blocked the visual manipulation of whether the hand or an object was viewed, and hence did not resolve the issue. Identifying the time taken for viewing one's own body to influence touch clearly requires manipulating visual context as a discrete visual event, rather than by prolonged blocks involving viewing the body or a neutral object for several successive trials.

Accordingly we have measured tactile acuity on the fingertip for somatosensory events during a dark interval that followed immediately after a brief, randomized glimpse either of the participant's hand, or of an object appearing at the same location. Vision of the hand or an object was unpredictably intermingled in an event-related manner. By continuously recording somatosensory evoked potentials, we investigated whether this rapid, unpredictable switching of visual context modulates somatosensory processing.

## 2 Methods

### 2.1 Participants

Thirty-three naïve, paid healthy volunteers (age 21–37, mean 24.2, 18 females) participated. All were right-handed as assessed by the Edinburgh Inventory (M: 81.6, range: 12.3–100). Data acquired from two further participants were excluded due to technical difficulties with EEG recording. Procedures were approved by the UCL research ethics committee and accorded with the principles of the Declaration of Helsinki.

### 2.2 Stimuli and procedure

**Fig. 1:**
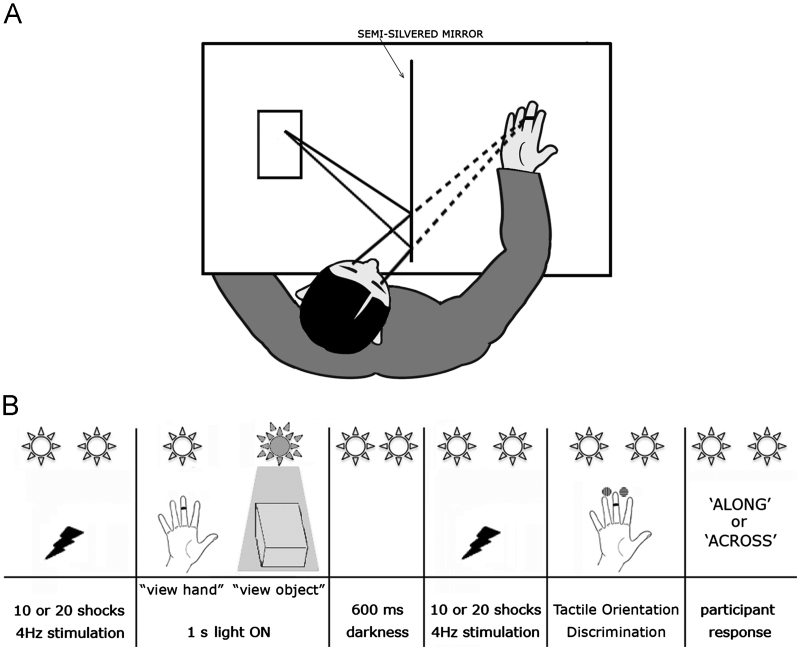
(A) Schematic depiction of experimental setup. Depending on illumination, participants either saw their right hand behind the semi-silvered mirror (dashed lines), or saw a reflection of a neutral object placed in front of the mirror (solid lines). (B) Schematic depiction of an experimental trial. In complete darkness participants were electrically stimulated several times on the right middle finger. Then either the hand or object was illuminated for 1 s at random. After illumination a second train of shocks was presented. Finally, a robot applied an oriented tactile grating to the middle finger, and participants verbally reported grating orientation.

Participants sat in complete darkness with their right arm resting palm-up on a table and looked into a semi-silvered mirror aligned with their parasagittal plane. Their right hand was positioned behind the mirror, while a hand-size wooden block (henceforth referred to as the ‘object’) was placed in front of the mirror (Fig. 1(A)). Two computer-controlled LED arrays were suspended behind the mirror above the hand, and in front of the mirror above the object, respectively. When the LED array behind the mirror was illuminated, the mirror functioned as a window and participants saw their right hand. When the LED array in front of the mirror was illuminated, participants saw instead the wooden object appearing at the hand's location. Previous studies using these mirror-box techniques (Longo et al., 2009; Longo, Musil, & Haggard, 2012b; Mancini, Longo, Kammers, & Haggard, 2011; Ramachandran, Rogers-Ramachandran, & Cobb, 1995) confirmed that they successfully induce illusions of location, so that the object in front of the mirror is perceived to be behind the mirror. Participants were asked to focus visual attention and gaze directly towards the location of the hand/object in all conditions. We controlled for spatial attention by ensuring that the hand and the object were actually seen in the same spatial location. To this aim before the experimental session the experimenter verified their perceived spatial location both on an individual level and also by monitoring the presence of any vertical or horizontal eye movements in the EEG recording as soon as one or the other light was switched on. In case any eye movement was shown, the objects location was re-adjusted and eye movements were checked again by repeating the previously described procedure.

Electrical stimulation was delivered through ring electrodes placed over the distal phalanx of the right middle finger. A neurophysiological stimulator provided a square-wave pulse for 0.2 ms, at an intensity 1.4 times each participant's sensory detection threshold as measured by an initial staircase procedure (Cornsweet, 1962), as follows. Briefly, participants were asked to report the presence or absence of the electrical stimulus delivered to the finger by verbal ‘yes’ or ‘no’ responses. Shock intensity began at 0 mA increasing in steps of 10 mA until the participant reported the presence of the stimulus. If the participant responded ‘yes’ three times consecutively, the shock intensity was reduced by 5 mA. If they responded ‘no’, intensity was increased. Progressively smaller changes were made until the participant was able to detect between 55% and 60% of shocks delivered to the finger. The mean threshold was 54 mA (SD 18 mA). These parameters ensured that electric stimulation corresponded to the same somatotopic location as the tactile perception task (see later), but did not interfere with tactile perception.

On each experimental trial, participants first received a train of either 10 or 20 electrical stimulations at 1.4*x* threshold in darkness. Electrical stimuli were delivered at 4 Hz. This relatively high stimulation rate was chosen to allow enough trials in each condition to produce a clear ERP average despite the relatively weak stimulation. Then, one of the two LED arrays selected at random illuminated the hand or object for 1 s. 600 ms after illumination ceased, a further train of either 10 or 20 shocks was presented in darkness. Finally, a tactile grating (Van Boven & Johnson, 1994) was applied by a robotic apparatus to the right middle fingertip. The number of electrical stimulations was randomly varied (10 or 20) to make the timing of vision and the timing of touch delivered by the robot unpredictable, thereby forcing participants to maintain attention continuously. There were 1040 stimuli in each experimental condition.

Tactile stimulation began immediately after the last shock, and lasted for 200 ms. Participants made unspeeded forced-choice verbal judgements regarding whether the grating ran along or across the finger (Fig. 1(B)). The tactile grating was selected to be just above each participant's discrimination threshold. The choice was based on an initial staircase procedure, using increasingly fine gratings to identify the smallest ridge-width for which accuracy was between 55% and 60% correct over 40 trials. The mean of the ridge widths selected by this approach was 1.01 mm (standard deviation=0.35 mm). During the staircase procedure, participants kept their eyes closed.

### 2.3 EEG recordings and analysis

A Neuroscan system (Neuroscan, El Paso, TX) was used to record EEG from electrodes placed at 17 standard scalp locations (FP1, FP2, F3, F4, C5, C3, Cz, C4, C6, CP5, CP3, CPz, CP4, CP6, O1, Oz, O2). The reference electrode was AFz and the ground electrode was placed on the chin. Electrode impedances were kept below 5 KΩ. The left and right mastoids were also recorded. Horizontal electroculogram (hEOG) was recorded from bipolar electrodes placed on the outer canthi of each eye, and vertical EOG (vEOG) was recorded from bipolar electrodes placed above and below the right eye. EEG signals were amplified and digitized at 1 KHz.

EEG data were analyzed with EEGLAB (Delorme & Makeig, 2004). Data were re-referenced to the average of the mastoids. Epochs were extracted from 50 ms before each finger shock to 350 ms after the shock trigger and the interval between 50 ms before the shock and the shock onset (0 ms) was used for baseline correction. A stimulation artifact 1–11 ms after the shock trigger was removed by linear interpolation. Data were low-pass filtered at 45 Hz. Trials with eyeblinks (where signal in any of FP1 and FP2, hEOG left and right, or vEOG up exceeded ±80 μV) or trials with signal exceeding ±120 μV in any channel were eliminated (mean 14% of trials, SD 11%). Grand averages were visually inspected to identify somatosensory event-related potential components. Well-known somatosensory event-related potential components were investigated (Allison et al., 1989a; Allison, McCarthy, Wood, Williamson, & Spencer, 1989b). Our interest focused on components associated with early somatosensory cortical processing (P50), and with somatosensory spatial attention (N140). Note that our 4 Hz stimulation rate meant that P50 potential for one shock would be superimposed on any P300 potential for the immediately preceding shock, so that these two ERPs could not be separated on purely temporal grounds. Therefore, we also investigated the P300 component. By comparing the form and scalp distribution of P50 and P300 ERPs to established patterns (Bruyant, Garcia-Larrea, & Mauguiere, 1993; Hamalainen, Kekoni, Sams, Reinikainen, & Naatanen, 1990), we could assess to what extent the P50 early cortical responses to one shock were contaminated by ‘cognitive’ P300 responses to the preceding shock. Peak amplitudes for each component were calculated by identifying maxima/minima in individual subject averages in each condition in the prototypical time window appropriate for each component as seen in the grand average (40–70 ms for the P50, 100–180 ms for the N140 and 290–330 ms for the P300).

We predicted an improvement in tactile orientation discrimination from viewing the hand, relative to viewing the object (Kennett et al., 2001). As regards somatosensory evoked potentials (SEPs), we predicted no differences between SEP components before visual exposure, since the view of hand or object has yet to occur. Crucially, if VET depends on rapid phasic integration of visual and tactile signals, we should predict a significant enhancement of somatosensory processing components after viewing the hand, relative to after viewing the object. Conversely, if VET reflects tonic changes in somatosensory activation occurring more slowly than the few seconds of our experimental trials, then no change in somatosensory processing components is predicted after viewing the hand.

## 3 Results

### 3.1 Behavioral Results

Judgments of grating orientation were significantly above chance both after viewing the hand (65% correct), [*t*_*(*32)_=7.98; *p*<0.0001] or the object (62% correct), [*t*_(32)_=5.39; *p*<0.0001]. More importantly, the difference between these conditions was significant: grating orientation discrimination was superior after briefly viewing the hand compared to after briefly viewing the object [*t*_(32)_=2.46; *p*<0.05, 2-tailed]. Thus a VET effect can be induced behaviorally by discrete, trial-specific glimpses of the hand, interspersed unpredictably with glimpses of the alternative object on other trials. Extended blocked viewing of the hand or object, as in prior studies of VET (Cardini et al., 2011) is not required.

### 3.2 Electrophysiological results

Scalp topographic maps showed early activity localized across contralateral central and parietal leads, corresponding to classical somatosensory cortices, whereas later activity more broadly distributed across the scalp.

**Fig. 2:**
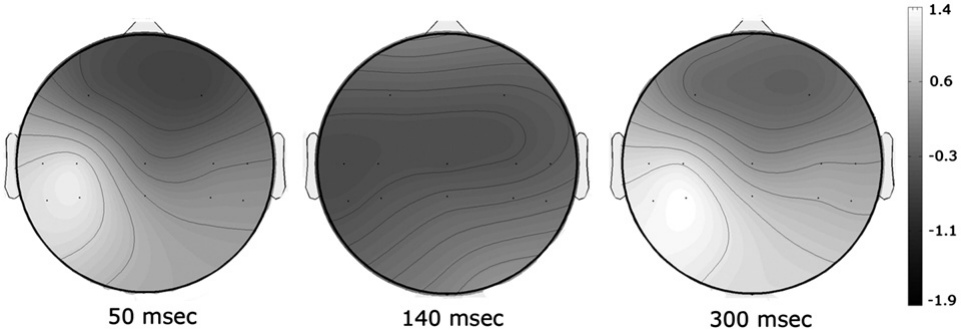
Grand average scalp maps across all conditions for the P50, N140 and P300 ERP components are shown (see discussion text for details).

Fig. 2 shows scalp topographic maps at three time points: at 50 ms, showing a strong positivity across the contralateral centro-parietal cluster (C3, C5, CP3, CP5 electrodes), which overlies the somatosensory cortex; a more broadly distributed negative activity across ipsilateral and contralateral centro-parietal sites (C3, C5, CP3, CP5, C4, C6, CP4, CP6 electrodes) at 140 ms; a broadly distributed positivity at around 300 ms. Three clear somatosensory components were identifiable from the grand averages: a P50 in the 40–70 ms time window, an N140 in the 100–180 ms time window and a P300 between 290 and 330 ms. The electrodes, which showed maximal deflection for each component, as listed above, were selected to investigate modulations of ERPs across visual conditions.

**Fig. 3:**
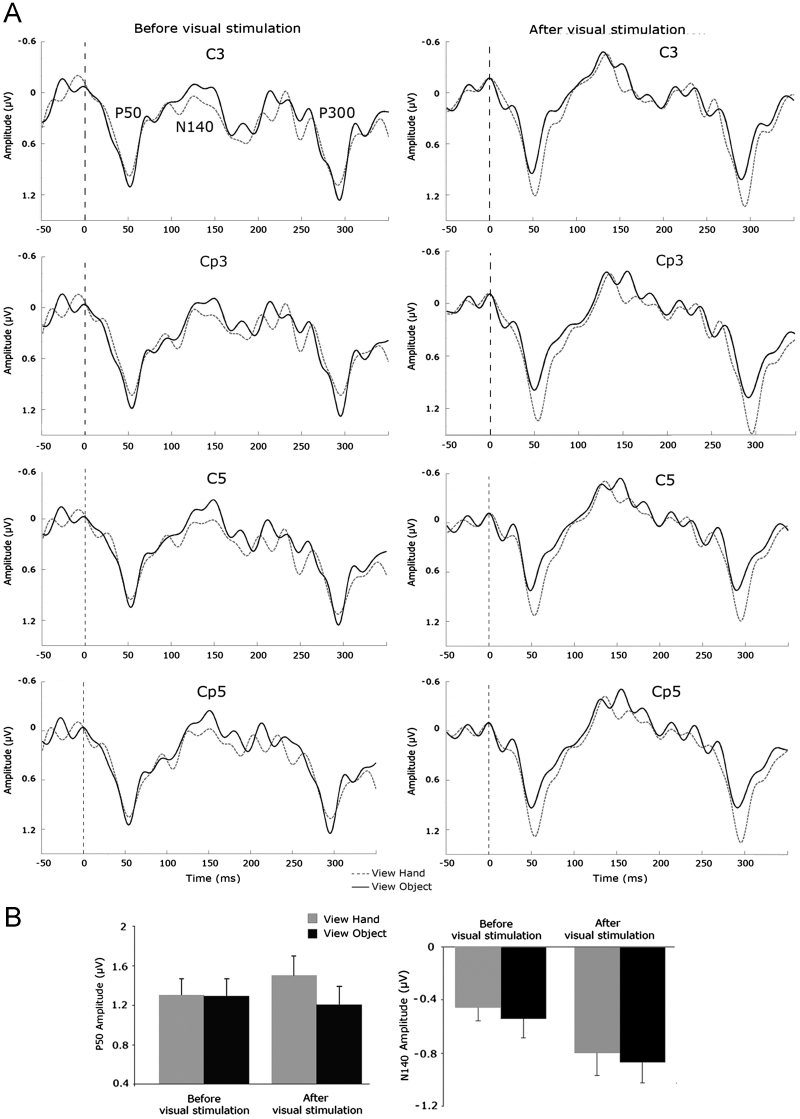
(A) Grand average ERP waveforms in the contralateral centro-parietal cluster (C3, C5, CP3, CP5) before and immediately after visual presentation of the hand (dashed line), and object (solid line). (B) Average of C3, C5, CP3 and CP5 P50 and N140 peak amplitudes in each condition, ± standard error.

We had a strong hypothesis that somatosensory stimulation and visual modulation would affect the SEP components recorded from the contralateral somatosensory cortex, based on both known anatomy and previous studies of SEP topography and time-course (Cardini et al., 2011). Therefore, peak amplitudes for each component in each condition were calculated and averaged across electrodes overlying the contralateral somatosensory cortex (C3, C5, CP3 and CP5). Component amplitudes were statistically analyzed using 2-by-2 ANOVAs with factors of view (hand vs object) and time (pre-vision vs post-vision). For P50 peak amplitude this revealed a main effect of view [*F*_(1,32)_=8.46; *p*<0.01], with higher amplitude for hand than for object, no main effect of time [*F*_(1,32)_=0.82; *p*=0.37]. More importantly, there was a significant view x time interaction [*F*_(1,32)_=4.34; *p*<0.05]. Follow-up t-tests showed that this interaction was due to an enhancement of P50 amplitude after glimpses of the hand. Specifically, we found a significant enhancement of P50 amplitude after the glimpse of the hand [*t*_(32)_=−2.19; *p*<0.05, 2-tailed] compared to before viewing the hand. No such enhancement, however, was found after viewing the object [*t*_(32)_=0.92; *p*=0.36, 2-tailed]. Comparing the hand and object conditions showed a significantly larger P50 after viewing the hand compared to after viewing the object [*t*_(32)_=3.01; *p*<0.01, 2-tailed], while P50 amplitudes were comparable before visual exposure [*t*_(32)_=0.13; *p*=0.89, 2-tailed] (see Fig. 3).

Similar analysis of N140 peak amplitude provided no evidence for visual modulation at this later stage of somatosensory processing. The 2-by-2 ANOVA revealed no effect of view [*F*_(1,32)_=1.41; *p*=0.24], a significant main effect of time [*F*_(1,32)_=13.38; *p*<0.01], and no significant interaction between these factors [*F*_(1,32)_=1.41; *p*=0.96]. In summary, N140 peak amplitude showed an overall enhancement after visual exposure relative to before, but this was independent of the visual context of what was seen.

Finally, comparable analysis of P300 peak amplitude provided no evidence for visual modulation specific to seeing the hand at this later stage of somatosensory processing. The 2-by-2 ANOVA did not show any significant main effects or interaction.

## 4 Discussion

Brief, unpredictable, and non-informative visual glimpses of the hand, randomly intermingled with other trials where an object was seen instead, enhanced tactile discrimination, and also facilitated early somatosensory processing of stimulation at the viewed skin location. The behavioural results extend previous reports of VET obtained in paradigms where participants continuously viewed the hand across repeated trials of a block. Here, we randomly interspersed brief 1 s glimpses of hand or object, and showed that viewing one's own hand can influence tactile acuity within a few seconds of visual onset. Moreover, somatosensory potentials showed that this brief vision of the body also affects tactile processing, again within a few seconds of visual onset.

We delivered shocks every 250 ms to produce reliable SEPs during our short experimental session. As a result, SEPs 50 ms after stimulus onset could represent a combination of P50 evoked by the immediately preceding shock and the P300 evoked by the shock before. The P50 arises in early somatosensory cortex, while the P300 is a late cognitive component. Therefore, understanding which of these components is modulated by brief glimpses of the body is important for the cognitive interpretation of our effects. Classical P50 and P300 components have quite different form and scalp topography. The P50 is maximal over contralateral somatosensory areas (Allison et al., 1989a; Ishibashi et al., 2000; Mauguiere, Desmedt, & Courjon, 1983), and characteristically shows reversal across the central sulcus. The classical somatosensory P300 is a very broad peak, with a broad scalp topography including frontal, parietal and temporal sites (Kida, Nishihira, Hatta, & Wasaka, 2003). It is generally bilateral, at least at central and parietal sites (Bruyant et al., 1993; Desmedt & Tomberg, 1989).

We could therefore use these classical forms to interpret the observed P50 and P300 SEPs in our data. In particular, we could assess whether our P50 component was contaminated by P300, or rather our P300 component was contaminated by P50. Fig. 2 shows the scalp topography of the P50 and P300 components in our data, averaged across subjects and conditions. The P50 component shows the contralateral focus and reversal across the central sulcus characteristic of early somatosensory components (Allison et al., 1989b; Hamalainen et al., 1990). Interestingly, our P300 did not show the broad distribution of the classical P300, but instead showed a focus similar to our P50. These observations suggest that our P300 components may be contaminated by P50 from the subsequent shock, but provide little evidence for the reverse effect of P50 contamination by P300 from the previous shock. This impression is confirmed by the width of the components (see Fig. 3): our P50 component showed the narrow peak of the classical P50. In contrast, our P300 did not show the broad peak of the classical P300, but rather a very narrow peak similar to our P50.

Our design involved a constant, high rate of somatosensory stimulation to ensure enough trials to generate a clear ERP in a single brief experiment. As a corollary, we cannot exclude some overlap of ERP components, and should be cautious in identifying which specific ERP components show rapid modulation from a brief glimpse of the hand. However, for all the reasons given above, we believe that our results provide evidence of rapid visual modulation of early stages of tactile processing, notably the P50 component arising in early somatosensory cortex.

In contrast, as far as the second somatosensory wave observed in the present data is concerned, no view-specific modulation of the later N140 component was found. Instead this later component was increased nonspecifically following glimpses of either hand or object. The N140 component is known to be sensitive to general attentional factors (Ohara, Lenz, & Zhou, 2006), and may involve frontal responses driven by somatosensory inputs (Allison, McCarthy, & Wood, 1992).

This study was designed to investigate whether VET can arise phasically following a specific visual event of viewing the body, or only more tonically over the more extended timescales of neuroplastic changes. We found that visual enhancement of touch can arise within a few seconds of viewing the body, and probably influences early somatosensory processing over the same timescale. Specifically, we found enhanced SEPs to trains of somatosensory stimuli beginning 1.6 s after the onset of viewing the hand, and immediately followed by tactile stimulation. Previous studies had reported effects of viewing the body on *early* somatosensory components (Cardini et al., 2011; Forster & Eimer, 2005; Taylor-Clarke et al., 2002). However, those studies used blocked designs, in which the hand or object was viewed continuously over an extended period. Such studies cannot clarify the time taken for viewing a body part to influence somatosensory processing. The present result is the first, to our knowledge, to address this question. Our results suggest that VET may occur much more rapidly than the neuroplastic changes involved in use-dependent intersensory substitution (Merabet & Pascual-Leone, 2010), in use-dependent somatosensory plasticity (Godde, Spengler, & Dinse, 1996), or in learning of multisensory associations (Zhou & Fuster, 2000).

Instead, we suggest VET may reflect a special form of phasic multisensory integration, which we call *contextual* integration. Previous studies of multisensory integration emphasised rapid feed-forward integration of visual and tactile information about the same external object, both subcortically (Stein & Meredith, 1993) and cortically (Avillac et al., 2007). In human multisensory perception, visual and haptic information must be present concurrently, and perceptually bound to the same object, for such feed-forward integration to occur efficiently (Helbig & Ernst, 2007). In contrast, our hand and object visual stimuli were both non-informative about the critical tactile events in our study, ruling out explanations based on binding events in separate modalities to form a single perceptual object (Driver, 1996). Moreover, our randomized design with discrete visual events and intermingled trial-types should prevent any continuous build-up of consistent multisensory associations (Zhou & Fuster, 2000) within our experiment. Thus, VET cannot simply reflect feed-forward integration of simultaneous visual and tactile input about the *same* multisensory event, nor can it reflect accumulating association between a visual stimulus and somatosensory information.

Rather, viewing a body part could rapidly activate a representation of that body part and/or the peripersonal space around it. Several studies confirm that such a multisensory, higher-order representation of the body exists in parietal and premotor association cortices (Gentile, Petkova, & Ehrsson, 2011). Recurrent projections from these representations could then provide a top-down modulating influence on early somatosensory cortex (Longo et al., 2012a). This would allow a brief glimpse of the body to influence somatosensory processing beginning rapidly after visual onset, and with effects lasting after the activating visual input is removed. VET may therefore provide an example of a contextual influence on sensory processing, rather than feed-forward integration between two sensory inputs about the same external event. Here we show that VET can emerge rapidly from discrete visual context events, over a timescale of seconds, rather than requiring plastic changes over several minutes. Future research could reveal the lower bound for its operation, by identifying the shortest visual-somatosensory intervals at which VET occurs.

The VET effects reported here are not readily explained by mere cross-modal links in spatial attention (Kennett, Spence, & Driver, 2002; Spence, Pavani, & Driver, 2000). We controlled spatial attention by ensuring that both hand and object were always viewed at exactly the same location. One recent study used an elegant factorial design to dissociate effects of viewing the hand from effects of gazing in the direction of the hand (Forster & Eimer, 2005). The results suggested that gaze acted as a modulator of spatial attention, affecting primarily the N140. In contrast, vision of the hand affected the earlier P50 component, as also found here. Our findings add the important information that P50 enhancement due to VET emerges quite rapidly, after only a brief glimpse of the hand, and then persists during a subsequent dark interval.

Our results also showed a significant enhancement of the N140 after viewing either hand or object, compared to before visual exposure. This could reflect either non-specific alerting effects of visual exposure on somatosensory processing, or a visual-tactile link in spatial attention. Specifically, visual stimulation could have exogenously enhanced tactile attention at the corresponding location, in accord with the well-known susceptibility of the N140 to attention (Nakajima & Imamura, 2000). However, we found different sensitivity to visual exposure and to visual content for the P50 and N140. This further underlines the distinction between effects of visual spatial attention, versus the more specific effects of viewing the body as identified here.

Multisensory enhancement, and visual enhancement of touch in particular, have clear adaptive value. VET facilitates processing of tactile events on one's own body. As soon as a body part is seen, modulation of corresponding primary somatosensory cortex may serve to enhance object perception on the body surface. Previous studies in humans (Ladavas, di Pellegrino, Farne, & Zeloni, 1998; Makin, Holmes, & Zohary, 2007) and primates (Colby, Duhamel, & Goldberg, 1993; Duhamel, Colby, & Goldberg, 1998) confirm that the parietal cortex, as well as other areas, maintains a multisensory representation of the body and of the space surrounding it. However, the functions of this representation are not yet fully clear. Coordination of grasping movements and defensive responding to potentially threats to the body surface have been suggested (Graziano & Cooke, 2006). Our results suggest that these multisensory representations may also modulate unisensory processing.
